# Thermoregulation and golden hour practices in extremely preterm infants: an international survey

**DOI:** 10.1038/s41390-022-02297-0

**Published:** 2022-09-08

**Authors:** Pranav Jani, Umesh Mishra, Julia Buchmayer, Karen Walker, Duygu Gözen, Rajesh Maheshwari, Daphne D’Çruz, Krista Lowe, Audrey Wright, James Marceau, Mihaela Culcer, Archana Priyadarshi, Adrienne Kirby, James E. Moore, Ju Lee Oei, Vibhuti Shah, Umesh Vaidya, Abdelmoneim Khashana, Sunit Godambe, Fook Choe Cheah, Wenhao Zhou, Hu Xiaojing, Muneerah Satardien

**Affiliations:** 1 Department of Neonatology, Westmead Hospital, Westmead, NSW Australia; 2 The University of Sydney, Sydney, NSW Australia; 3 Comprehensive Center for Pediatrics, Department of Pediatrics and Adolescent Medicine, Division of Neonatology, Pediatric Intensive Care and Neuropediatrics, Medical University of Vienna, Vienna, Austria; 4 Department of Newborn Care, Royal Prince Alfred Hospital, Camperdown, NSW Australia; 5 Council of International Neonatal Nurses, Boston, MA USA; 6 The George Institute for Global Health, Sydney, NSW Australia; 7 Sydney Institute for Women, Children & their Families, Sydney, NSW Australia; 8 Pediatric Nursing Department, Florence Nightingale Faculty of Nursing, İstanbul University – Cerrahpaşa, İstanbul, Turkey; 9 The National Health and Medical Research Council Clinical Trials Centre, University of Sydney, Sydney, NSW Australia; 10 Connecticut Children’s, Division of Neonatal-Perinatal Medicine, Connecticut Children’s Medical Center, Hartford, CT USA; 11 UCONN School of Medicine Farmington, Farmington, CT USA; 12 The Royal Hospital for Women, Randwick, NSW Australia; 13 School of Women’s and Children’s Health, University of New South Wales, Randwick, NSW Australia; 14 Department of Paediatrics and Institute of Health Policy, Management and Evaluation, Mount Sinai Hospital, Toronto, ON Canada; 15 Department of Pediatrics, King Edward Memorial Hospital, Pune, India; 16 Pediatrics, Suez Canal University, Ismailia, Egypt; 17 Divisional Director for Clinical Governance, Women Children and Clinical Support, Imperial College Healthcare NHS Trust, London, UK; 18 Department of Paediatrics, Faculty of Medicine, Universiti Kebangsaan Malaysia, Bangi, Malaysia; 19 Hospital Canselor Tuanku Muhriz, Jalan Yaacob Latif, Bandar Tun Razak, Kuala Lumpur, Malaysia; 20 Department of Neonatology and Vice President, Children’s Hospital of Fudan University, Shanghai, China; 21 Nursing Department, Children’s Hospital of Fudan University, Shanghai, China; 22 Department of Paediatrics and Child Health, Tygerberg Hospital Cape Town, Cape Town, South Africa; 23 University of Stellenbosch South Africa, Stellenbosch, South Africa

## Abstract

**Background:**

Are thermoregulation and golden hour practices in extremely preterm (EP) infants comparable across the world? This study aims to describe these practices for EP infants based on the neonatal intensive care unit’s (NICUs) geographic region, country’s income status and the lowest gestational age (GA) of infants resuscitated.

**Methods:**

The Director of each NICU was requested to complete the e-questionnaire between February 2019 and August 2021.

**Results:**

We received 848 responses, from all geographic regions and resource settings. Variations in most thermoregulation and golden hour practices were observed. Using a polyethylene plastic wrap, commencing humidity within 60 min of admission, and having local protocols were the most consistent practices (>75%). The odds for the following practices differed in NICUs resuscitating infants from 22 to 23 weeks GA compared to those resuscitating from 24 to 25 weeks: respiratory support during resuscitation and transport, use of polyethylene plastic wrap and servo-control mode, commencing ambient humidity >80% and presence of local protocols.

**Conclusion:**

Evidence-based practices on thermoregulation and golden hour stabilisation differed based on the unit’s region, country’s income status and the lowest GA of infants resuscitated. Future efforts should address reducing variation in practice and aligning practices with international guidelines.

**Impact:**

- A wide variation in thermoregulation and golden hour practices exists depending on the income status, geographic region and lowest gestation age of infants resuscitated.
- Using a polyethylene plastic wrap, commencing humidity within 60 min of admission and having local protocols were the most consistent practices.
- This study provides a comprehensive description of thermoregulation and golden hour practices to allow a global comparison in the delivery of best evidence-based practice.
- The findings of this survey highlight a need for reducing variation in practice and aligning practices with international guidelines for a comparable health care delivery.

## Introduction

The survival of extremely preterm (EP) infants (those born <28 weeks of gestation) has improved steadily due to advances in neonatal care.^1^ Many neonatal intensive care units (NICUs) are now providing active care to infants as young as 22 weeks gestation.^2^ One of the most critical periods of life is the “golden hour”, this refers to the first hour of life following the birth of an EP infant. During this important period, evidence-based initial stabilisation practices including maintenance of normothermia are performed. There is emerging evidence for better short-term outcomes from these practices, even in EP infants at borderline ages of viability.^3,4^

International resuscitation guidelines recommend the maintenance of normothermia (body temperature between 36.5 and 37.5 °C) from birth as a vital step in the stabilisation of preterm infants and list multiple strategies for thermoregulation.^5^ Implementation of these practices aims to prevent the occurrence of hypothermia and hyperthermia, both of which are associated with adverse outcomes.^6^ Regional variations exist for golden hour period practices.^7,8^ Whether NICUs around the world are comparable in their ability to deliver evidence-based practice is unknown. We designed this international survey to provide a comprehensive description of the variations in thermoregulation and golden hour practices for EP infants, categorised according to the NICUs' geographic location, country’s income status, and the lowest gestational age (GA) of infants resuscitated. We hypothesised that significant differences in thermoregulation and golden hour practices may be occurring. The findings of this study have implications for improving evidence-based practice depending on local availability and use of resources.

## Methods

A questionnaire was drafted, piloted, refined, and validated by the lead site’s research team. This international survey is an extension of the pilot study.^9^ Ethical approval was obtained from the Western Sydney Local Health District’s Human Research Ethics Committee (approval number LNR/18/WMEAD/288–5770) and by The Mount Sinai Hospital Research Ethics Board (approval number 20-0213-E). Research Electronic Data Capture (REDCap; Vanderbilt University, Nashville, TN) was used to generate a questionnaire. NICU directors were invited to participate by a direct email or by assistance from the regional professional neonatal or parent organisation. To increase participation in the survey, a reminder was sent twice after the initial request-to-participate invitation. Participation in the survey was voluntary and participants consented prior to completing the questionnaire. The invitation letter informed the participants about the time required to complete the survey, details on data storage and privacy of participants, details of the investigators, and the purpose of the study. The clinician most familiar with the participating NICU practices was requested to complete the survey. Questions were predominantly close-ended. Open-ended questions sought additional information where applicable, such as details on any commercial products used.

### Statistics

De-identified survey responses were collected, exported from REDCap to Stata 17 (Stata Corp, College Station, TX) and analysed. Descriptive statistics were used to summarise the responses. Responses are reported as numbers and percentages and are grouped based on the unit’s geographical region, the country’s income status (information from www.worldbank.org) and whether units were resuscitating infants who were born from 22 to 23 weeks or from 24 to 25 weeks GA. Binary logistic models were used to investigate the relationship between the lowest GA group of infants resuscitated and individual thermoregulation and golden hour practices adjusted by regional and income status group. Results from these models are reported with odds ratios and 95% confidence intervals. In addition, we have reported two-tailed *p* value <0.05 as significant.

## Results

**Fig. 1:**
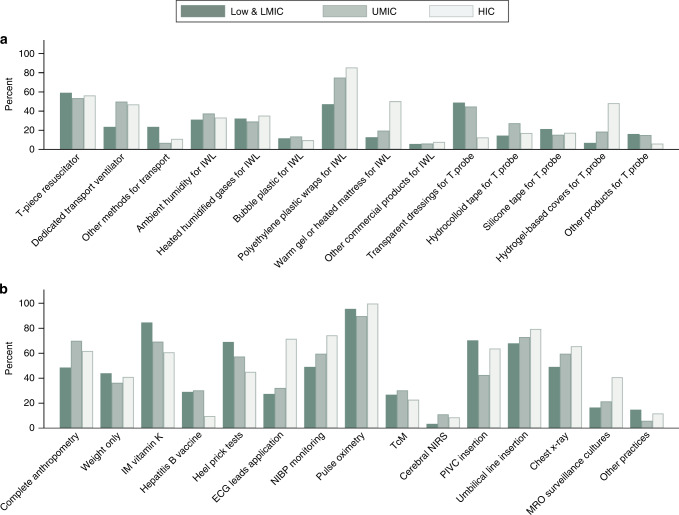
Thermoregulation and golden hour practices based on income status groups. Thermoregulation practices (**a**) and golden hour practices (**b**) by income status groups of the respondent units. IWL insensible water losses, T.probe temperature probe, IM intramuscular, ECG electrocardiogram, NIBP non-invasive blood pressure, TcM transcutaneous monitoring, NIRS near infra-red spectroscopy, PIVC peripheral intravenous catheter, MRO multi-resistant organisms, Low and LMIC low and lower-middle-income country, UMIC upper-middle-income country, HIC high-income country.

**Fig. 2:**
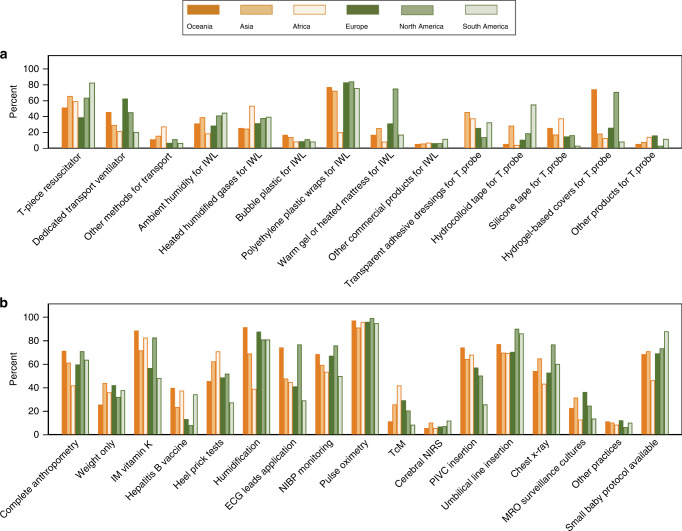
Thermoregulation and golden hour practices based on geographic regions. Thermoregulation practices (**a**) and golden hour practices (**b**) by geographic region. IWL insensible water losses, T.probe temperature probe, IM intramuscular, ECG electrocardiogram, NIBP non-invasive blood pressure, TcM transcutaneous carbon dioxide monitoring, NIRS near infra-red spectroscopy, PIVC peripheral intravenous catheter, MRO multi-resistant organisms.

A total of 848 responses were received (shown in Table 1), and respondents were from 100 countries (Supplementary Material 1). Responses based on income status group are shown in Table 2 and Fig. 1. Responses were obtained from six geographic regions (shown in Table 3 and Fig. 2). The questionnaire was completed by 60% (*n* = 507) medical staff and 40% (*n* = 339) nursing staff; two were undefined. The lowest GAs from which resuscitation was offered were 22 weeks (29%), 23 weeks (32%), 24 weeks (22%) and 25 weeks (17%). This varied according to income status groups and geographic regions (shown in Tables 2 and 3).

**Table 1:** Thermoregulation and golden hour practices for all respondent units.

Details on practices	Proportion of respondent’s number (%)
*Practices in the birthing environment (n* *=* *respondents who answered the question)*	
Using heated humidified gases at resuscitation (*n* = 813)	394 (48)
Method of transporting preterm infants from the birthing environment, multiple choices allowed (*n* = 848)	
T-piece resuscitator CPAP/IPPV	475 (56)
Dedicated transport ventilator	364 (43)
Both	104 (12)
Others	103 (12)
Methods to minimise IWL, multiple choices allowed (*n* = 816)	
Ambient humidity	292 (36)
Heated humidified gases at birth	277 (34)
Bubble plastic	95 (12)
Polyethylene occlusive plastic wraps	628 (77)
Warm gel mattress or heated mattress	277 (34)
Other commercial products	58 (7)
Methods to secure the skin temperature probe, multiple choices allowed (*n* = 751)	
Transparent adhesive dressings	259 (35)
Hydrocolloids	167 (22)
Silicone-based tapes	147 (20)
Hydrogel-based covers	254 (34)
Any other product	94 (13)
Site of securing the skin temperature probe at resuscitation (*n* = 805)	
Axillae	115 (14)
Front of abdomen	567 (70)
On the back	88 (11)
Any other site	35 (4)
Heating mode used during transport of infants (*n* = 765)	
Manual	403 (53)
Servo control	362 (47)
Ability to change the environmental ambient temperature of the birthing place (*n* = 824)	506 (61)
Ambient birthing environmental temperature (*n* = 733)	
Cold (<24 °C)	107 (14)
Normal (24–26 °C)	309 (42)
Warm (>26 °C)	181 (25)
Cold to normal (<24–26 °C)	28 (4)
Normal to warm (24–>26 °C)	25 (3)
*Practices at admission to the NICU*	
Golden hour practices at admission, multiple choices allowed (*n* = 845)	
Complete anthropometry (weight, length, head circumference)	519 (62)
Weight measurement only	335 (40)
Intramuscular vitamin K injection	574 (68)
Hepatitis B vaccine	171 (20)
Heel prick for blood sugar/capillary blood gas	454 (54)
Commence humidification	650 (77)
ECG lead application	419 (50)
NIBP cuff application	540 (64)
Pulse oximetry	803 (95)
TcM if ventilated	218 (26)
Cerebral oximetry using NIRS	71 (8)
Peripheral intravenous cannulation	490 (58)
Umbilical line insertion	630 (75)
Chest x-ray	506 (60)
Infection control surveillance cultures from skin	247 (29)
Other monitoring or intervention performed	88 (10)
Commencement of humidity upon admission (*n* = 792)	
Within 60 min	690 (87)
1–3 h	88 (11)
3–6 h	6 (1)
Beyond 6 h	8 (1)
Level of humidity at commencement (*n* = 790)	
60–70%	179 (23)
71–80%	247 (31)
81–90%	257 (33)
>90%	107 (14)
A local small baby protocol available (*n* = 791)	591 (75)
A local thermoregulation guideline (*n* = 828)	748 (90)
A local ambient humidity guideline (*n* = 807)	624 (77)

Responses to questions reported as column number (%), percentage rounded to the nearest whole number.

*CPAP* continuous positive airway pressure, *IPPV* intermittent positive pressure ventilation, *IWL* insensible water losses, *ECG* electrocardiogram, *NIBP* non-invasive blood pressure, *TcM* transcutaneous monitoring, *NIRS* near infra-red spectroscopy.

**Table 2:** Practices based on income status groups of the respondent units.

Practices	Low and LMIC 175 (21)	UMIC 275 (33)	HIC 392 (47)
*Practices in the birthing environment (n* *=* *respondents who answered the question)*			
Lowest GA of infants offered resuscitation (*n* = 841)			
22 weeks	15 (9)	75 (27)	150 (38)
23 weeks	16 (9)	67 (24)	188 (48)
24 weeks	63 (36)	72 (26)	51 (13)
25 weeks	80 (46)	61 (22)	3 (1)
Using heated humidified gases at resuscitation (*n* = 807)	74 (45)	157 (61)	160 (42)
Site of securing the skin temperature probe at resuscitation (*n* = 800)			
Axilla	13 (8)	31 (11)	71 (20)
Front of abdomen	146 (86)	210 (78)	207 (58)
On the back	9 (5)	24 (9)	55 (15)
Other sites	2 (1)	5 (2)	27 (8)
Heating mode used during transport of infants (*n* = 761)			
Manual mode	74 (52)	116 (44)	212 (60)
Servo mode	69 (48)	147 (56)	143 (40)
Ability to change the environmental ambient temperature of the birthing place (*n* = 819)	100 (58)	155 (59)	247 (64)
Ambient birthing environmental temperature (*n* = 730)			
Cold (<24 °C)	7 (5)	75 (31)	107 (32)
Normal (24–26 °C)	54 (35)	111 (46)	142 (42)
Warm (>26 °C)	76 (49)	33 (14)	72 (21)
Cold to normal (<24–26 °C)	3 (2)	16 (7)	9 (3)
Normal to warm (24–>26 °C)	14 (9)	4 (2)	7 (2)
*Practices at admission to the NICU*			
Commencement of humidity upon admission (*n* = 786)			
Within 60 min	120 (87)	236 (91)	329 (85)
1–3 h	17 (12)	16 (6)	55 (14)
3–6 h	1 (1)	2 (1)	3 (1)
Beyond 6 h	0	6 (2)	1 (0.26)
Level of humidity at commencement (*n* = 785)			
60–70%	53 (39)	72 (27)	53 (14)
71–80%	54 (40)	66 (25)	125 (32)
81–90%	16 (12)	99 (38)	140 (36)
>90%	12 (9)	27 (10)	68 (18)
A local small baby protocol available (*n* = 786)	110/162 (68)	203/261 (78)	274/363 (75)
A local thermoregulation guideline (*n* = 822)	157/171 (92)	234/266 (88)	351/385 (91)
A local ambient humidity guideline (*n* = 801)	81/164 (49)	211/260 (81)	326/377 (86)

Six respondents did not identify their country. Responses reported as column number (%), percentage rounded to the nearest whole number.

*GA* gestational age, *Low and LMIC* low and lower-middle-income country, *UMIC* upper-middle-income country, *HIC* high-income country, *NICU* neonatal intensive care unit.

**Table 3:** Practices based on the geographic region of the respondent units.

Practices	Europe (*n* = 300)	Asia (*n* = 259)	N. America (*n* = 121)	Africa (*n* = 69)	S. America (*n* = 58)	Oceania (*n* = 35)
*Practices in the birthing environment (n* *=* *respondents who answered the question)*						
Lowest GA (*n* = 841)						
22 weeks	112 (37)	41 (16)	63 (52)	6 (9)	15 (26)	3 (9)
23 weeks	119 (40)	65 (25)	35 (29)	3 (4)	23 (40)	26 (74)
24 weeks	59 (20)	79 (31)	7 (6)	21 (30)	15 (26)	5 (14)
25 weeks	10 (3)	73 (28)	16 (13)	39 (57)	5 (9)	1 (3)
Using heated humidified gases at resuscitation (*n* = 807)	159 (54)	108 (44)	51 (43)	29 (47)	32 (57)	12 (36)
Site of securing the skin temperature probe at resuscitation (*n* = 800)						
Axilla	37 (13)	32 (13)	29 (24)	8 (12)	2 (4)	7 (21)
Front of abdomen	175 (62)	181 (75)	84 (69)	53 (82)	49 (88)	21(64)
On the back	47 (17)	26 (11)	3 (2)	4 (6)	5 (9)	3 (9)
Other sites	24 (8)	3 (1)	5 (4)	0	0	2 (6)
Heating mode used during transport of infants (*n* = 761)						
Manual mode	145 (53)	120 (51)	50 (44)	30 (53)	35 (66)	22 (71)
Servo mode	128 (47)	114 (49)	63 (56)	27 (47)	18 (34)	9 (29)
Ability to change the environmental ambient temperature of the birthing place (*n* = 819)	163 (55)	160 (64)	87 (74)	28 (42)	46 (81)	18 (56)
Ambient birthing environmental temperature (*n* = 730)						
Cold (<24 °C)	80 (30)	34 (15)	55 (54)	1 (2)	6 (11)	13 (45)
Normal (24–26 °C)	109 (41)	105 (47)	34 (34)	13 (22)	32 (59)	14 (48)
Warm (>26 °C)	54 (20)	64 (29)	7 (7)	43 (74)	12 (22)	1 (3)
Cold to normal (<24–26 °C)	16 (6)	5 (2)	3 (3)	0	4 (7)	0
Normal to warm (24–>26 °C)	5 (2)	16 (7)	2 (2)	1 (2)	0	1 (3)
*Practices at admission to the NICU*						
Commencement of humidity upon admission (*n* = 786)						
Within 60 min	282 (95)	209 (90)	77 (66)	45 (87)	47 (85)	25 (76)
1–3 h	11 (4)	18 (8)	37 (32)	7 (13)	7 (13)	8 (24)
3–6 h	1 (0.34)	2 (1)	2 (2)	0	1 (2)	0
Beyond 6 h	3 (1)	3 (1)	1 (1)	0	0	0
Level of humidity at commencement (*n* = 785)						
60–70%	54 (18)	58 (25)	39 (33)	24 (47)	0	3 (9)
71–80%	94 (32)	66 (29)	45 (38)	23 (45)	7 (13)	10 (31)
81–90%	113 (38)	56 (24)	25 (21)	4 (8)	41 (73)	16 (50)
>90%	35 (12)	51 (22)	10 (8)	0	8 (14)	3 (9)
A local small baby protocol available (*n* = 786)	207/279 (74)	184/237 (78)	89/116 (77)	32/66 (48)	51/55 (93)	24/33 (73)
A local thermoregulation guideline (*n* = 822)	250/292 (86)	226/253 (89)	113/120 (94)	64/67 (96)	56/56 (100)	33/34 (97)
A local ambient humidity guideline (*n* = 801)	228/284 (80)	181/243 (74)	108/119 (91)	20/65 (31)	49/56 (88)	32/34 (94)

Responses reported as column number (%), percentage rounded to the nearest whole number.

*N. America* North America, *S. America* South America.

### Thermoregulation practices in the birthing environment

#### Heated humidified gases and the method of transport

Heated humidified gases were used during resuscitation in 394 units (48%). More units (61%) from upper-middle-income countries (UMIC) were using them than units (45%) from low and lower-middle-income countries (Low and LMIC), and high-income countries (HIC) (42%) (shown in Table 2). Units from South America used these the most (57%), and units from Oceania (36%) used these the least (shown in Table 3). A T-piece resuscitator was the most common device used when transporting infants from the birthing unit to the NICU (56%). The proportion of units using a T-piece resuscitator based on the income status groups was similar, but a greater proportion of UMIC (50%) and HIC (47%) units used a dedicated transport ventilator than low and LMIC (23%) units (shown in Figs. 1 and 2). Regional variation in the use of T-piece resuscitators and dedicated transport ventilators was also observed.

#### Minimising insensible water losses (IWL)

A polyethylene occlusive plastic wrap was used most often for minimising IWL (77%). Variation in its use was observed based on the unit’s income status group. African units used a polyethylene occlusive plastic wrap the least (20%) compared to >70% for units from all other regions. A warm gel or heated mattress was used more often in units from HIC, 50% as compared to <20% for units from the other two income status groups. Units from North America used a warm gel or heated mattress (75%) the most. Overall, nearly a third of the units used more than one strategy for minimising IWL.

#### Temperature-probe placement at resuscitation

Transparent adhesive dressing and hydrogel-based covers were the two most common methods for securing the temperature probe at resuscitation (35 and 34%, respectively, shown in Table 1). A transparent dressing was more frequently used in units from low and LMIC (49%) and UMIC (45%) and hydrogel-based covers were most often used in units from HIC (48%). A geographic variation in the method of securing the temperature probe was observed. A transparent dressing was most often used in Asia (46%), hydrocolloid dressing in South America (55%), silicone-based tapes in Africa (38%), and hydrogel-based covers in Oceania (74%) and North America (71%). The front of the abdomen was the most common site for temperature-probe placement (70%). A greater proportion of units from HIC secured the temperature probes in the axilla (20%) and on the back (15%) than units in the other two income groups (shown in Table 2).

#### Environmental temperature and transport

There was great variation in the environmental temperature with only 42% units reporting an ambient birthing environmental temperature of 24–26 °C (shown in Table 1). Nearly three-quarters of the units from Africa had an ambient birthing environmental temperature >26 °C whereas units in Oceania (45%) and North America (54%) had an ambient birthing environmental temperature <24 °C (shown in Table 3). Most units were able to change the ambient temperature of the birthing place (shown in Table 1); the lowest use of this practice was in Africa (42%) and the highest in South America (81%) shown in Table 3.

Servo control and manual modes were used approximately equally to provide heat during the transport of infants from the birthing place to the NICU (shown in Table 1). A manual mode was used more in units from HIC (60%) than units from UMIC (44%) shown in Table 2. All geographic regions except for North America (44%) used the manual mode to a similar extent (>50%) shown in Table 3.

### Golden hour practices at admission to the NICU

A complete anthropometry was performed by 62% units, and nearly two-thirds of units administered a vitamin K injection (shown in Table 1). Pulse oximetry was the most consistent monitoring, followed by non-invasive blood pressure (NIBP) monitoring, application of electrocardiogram (ECG) leads, transcutaneous carbon dioxide for ventilated patients and cerebral oximetry. Inserting umbilical catheters (75%) was the preferred practice over peripheral intravenous catheters (58%) shown in Table 1. Administration of a vitamin K injection, hepatitis B vaccine, a heel prick for blood tests, and peripheral intravenous catheter insertion were more common practices in low and LMIC units, whereas using ambient humidity, application of ECG leads, NIBP monitoring, insertion of umbilical catheters, performing a chest x-ray, and collecting multi-resistant organism surveillance swabs were the most common practices for units from HIC (shown in Fig. 1). A geographic variation in golden hour practices was also observed for heel prick tests, the use of ambient humidity, ECG lead application, transcutaneous carbon dioxide monitoring, and peripheral intravenous catheter use (shown in Fig. 2). Most units (87%) commenced ambient humidity within 60 min of admission, but the level of humidity varied from 60 to >90% (shown in Table 3). This variation was also found based on the income status groups and the geographic regions of the units. Overall, 75% units had a small baby protocol (comprehensive local guideline for the management of EP infants); however, only 48% units from Africa had this protocol (shown in Table 3).

Selected thermoregulation and golden hour practices for units resuscitating infants from 22 to 23 weeks GA differed significantly from units resuscitating infants from 24 to 25 weeks GA. They were more likely to use a dedicated transport ventilator for patient transport, a polyethylene plastic wrap to reduce IWL in the birthing environment, use higher levels of ambient humidification (>80%), and have local clinical protocols (shown in Table 4).

**Table 4:** Relationships between lowest gestational age at resuscitation and delivery room practices.

Practices	Resuscitating from 22 to 23 weeks (*n* = 515)	Resuscitating from 24 to 25 weeks (*n* = 332)	aOR (95% CI^a^)	*p* value
*Practices in the birthing environment (n* *=* *respondents who answered the question)*				
Using heated humidified gases at resuscitation (*n* = 812)	246/498 (49)	147/314 (47)	0.71 (0.50–1.01)	0.06
Method of transporting preterm infants from the birthing environment (*n* = 847)				
T-piece resuscitator	273 (53)	201 (61)	1.50 (1.07–2.11)	0.01
Dedicated transport ventilator	258 (50)	106 (32)	0.54 (0.39–0.77)	0.001
Methods to minimise IWL (*n* = 847)				
Ambient humidity	187 (36)	104 (31)	0.77 (0.54–1.10)	0.15
Heated humidified gases at birth	181 (35)	96 (29)	0.76 (0.53–1.09)	0.14
Bubble plastic	56 (11)	39 (12)	0.91 (0.54–1.53)	0.74
Polyethylene occlusive plastic wraps	424 (82)	204 (61)	0.66 (0.44–0.97)	0.03
Warm gel mattress or heated mattress	210 (41)	67 (20)	0.81 (0.55–1.19)	0.29
Heating mode used during transport of infants (*n* = 764)				
Servo control	202 (42)	160 (56)	1.60 (1.12–2.27)	0.008
Ability to change the environmental ambient temperature of the birthing place (*n* = 823)	327/504 (65)	178/319 (56)	0.84 (0 62–1.12)	0.25
*Practices at admission to the NICU*				
Commencement of humidity upon admission (*n* = 792)				
Within 60 min	429 (85)	260 (90)	1 (base)	
Beyond 60 min	74 (15)	28 (10)	0.65 (0.14–1.14)	0.14
Level of humidity at commencement (*n* = 789)				
60–70%	81 (16)	97 (34)	1 (base)	
71–80%	152 (30)	95 (33)	0.66 (0.42–1.06)	0.09
81–90%	187 (37)	70 (25)	0.54 (0.34–0.87)	0.01
>90%	83 (17)	24 (8)	0.36 (0.19–0.68)	0.002
A local small baby protocol available (*n* = 790)	378/482 (78)	212/308 (69)	0.59 (0.40–0.89)	0.01
A local ambient humidity protocol available (*n* = 806)	422/496 (85)	201/310 (65)	0.64 (0.42–0.98)	0.04
A local thermoregulation protocol available (*n* = 827)	462/507 (91)	285/320 (89)	0.76 (0.44–1.32)	0.33

Responses reported as column number (%), percentage rounded to the nearest whole number. Adjusted odds ratio from binary logistic regression models. Resuscitation from 22 to 23 weeks was arbitrarily used as the reference group.

*aOR* adjusted odds ratio, *CI* confidence interval, *IWL* insensible water losses.

^a^Models were adjusted for income status group and continents.

## Discussion

This is the first survey which comprehensively describes international thermoregulation and golden hour practices for EP infants. Differences in practices were observed based on the country’s income status, geographic location and the lowest GA of infants resuscitated.

Thermoregulation in EP infants is a challenge. These infants are at increased risk of serious complications from hypothermia and hyperthermia.^6,10,11^ Strategies suggested by international resuscitation guidelines to maintain normothermia in preterm infants include keeping the ambient temperature of the birthing environment above 23 °C, wrapping the infant in a polyethylene bag/wrap, using an exothermic heating mattress, and using heated humidified gases at resuscitation.^5,12^ In this survey, the use of a polyethylene bag or wrap was the most consistent practice for thermoregulation. This finding aligns with the current literature on interventions to prevent hypothermia at birth.^13^ The income status and geographic region may have influenced the methods used for reducing IWL. Further national and international efforts could be directed at improving the strength of evidence and resources to implement these interventions. It is known that the incidence of hypothermia can be reduced by increasing the temperature of the birthing place;^14^ however, this can often be difficult to implement and only 42% of responding units were able to change the ambient temperature. While a high ambient temperature could increase the risk of preterm birth,^15^ little is known about the effect of a high birthing-place temperature on neonatal outcomes for EP infants.

There is little evidence about the optimal site of securing a skin temperature probe and its effect on thermoregulation.^16^ We found variation in practice for the site of securing the probe. Most units preferred placing the temperature probe on the abdomen of the infant, but evidence to support this practice is lacking. Bensouda et al. recorded similar admission temperatures in moderate preterm infants from temperature-probe placement on the axilla, left upper and left lower back.^17^ A single centre randomised control trial in Australia is currently recruiting preterm infants <32 weeks gestation to ascertain the effect of temperature-probe placement in the axilla compared to the left upper back during delivery room resuscitation on admission body temperature (ACTRN12620000293965).

EP infants have physiological immaturity of the skin structures, which increases their risk of IWL especially in the first week of life.^18^ IWL is lower with the use of ambient humidity than with the use of a radiant heat warmer.^19^ We found that most units commenced humidity early upon admission, but there was variation in the level of humidity used, consistent with the previous literature.^20^ Further research is needed on the optimal starting level and duration of administration of humidity to streamline practice and allow meaningful analysis of outcomes.

Variation in practice is common, this may compromise the quality of patient care. This could reflect a local adaptation based on resource availability, a population health issue, or a lack of and/or ambiguity in the strength of available evidence. Developing and implementing an evidence-based protocol disseminates best practice locally.^21^ We identified variation in a local protocol availability based on the geographic region and on the lowest GA of infants resuscitated. This finding may assist units in creating and implementing a protocol that adapts to local health care challenges.

Resuscitating and managing preterm infants as young as 22–23 weeks GA is challenging. Interestingly, in our survey, many units were resuscitating infants from 22 to 23 weeks GA. Arbour et al. reported a variation in resource availability, possibly contributing to variation in clinical outcomes, for hospitals resuscitating EP infants.^22^ A recent survey from Europe identified many resuscitation practices aligned with the international newborn resuscitation guideline, however large variation in practice continued to occur.^23^ The current survey identifies differences in practices after adjusting for the continent and the income status groups for the lowest GA of infants resuscitated. Future research and quality improvement activities will bridge the gap in knowledge on the influence of bundles of best practices for stabilisation on clinical outcomes, especially for infants born at 22–23 weeks GA.

Large sample size and participation from all geographic regions and income status groups allow generalisability of the results. Although we surveyed tertiary NICU practices, the findings might be of relevance to a wider community of general paediatricians, nurses and policymakers who may be encountering the birth of EP infants at non-tertiary NICUs. We acknowledge the potential limitations of the study, recall bias was minimised by requesting the survey completion by the clinician most familiar with unit’s practices. The survey questionnaire was prepared only in English, which may have limited participation from non-English speaking countries. The COVID-19 pandemic may have affected the participation in the survey. An overall survey response rate was difficult to calculate and hence this was not reported for the following reasons: the information on the total number of NICUs from each participating region was not known, some regional organisations did not reveal this information, and inaccurate email addresses led to undelivered survey invitations. We did not collect information on other practices for resuscitation of EP infants as listed in international resuscitation guidelines. Data on the number of participating units from each country was not provided to maintain confidentiality. Finally, only one survey response per unit was collected, which may not allow exploration of practice variation especially where units did not have a local practice protocol.

## Conclusion

Using a polyethylene plastic wrap, commencing humidity within 60 min of admission, and having local protocols were the most consistent practices. Variation exists for most thermoregulation and golden hour practices based on the geographic region, country’s income status and the lowest GA of infants resuscitated. The findings of this study highlight a need for reducing variation in practice and for aligning practices to international guidelines. There is a need for improving the evidence on thermoregulation and golden hour practices for comparable health care delivery. These findings might affect future research, practice, resource availability and policy for the delivery of best evidence-based practice to EP infants.

## Supplementary information

Online supplementary material

## Data Availability

All data generated or analysed during this study are included in this published article. Region or country-specific data are available from the corresponding author on reasonable request.

## References

[1] Myrhaug, H. T., Brurberg, K. G., Hov, L. & Markestad, T. Survival and impairment of extremely premature infants: a meta-analysis. *Pediatrics* **143**, e20180933 (2019). 10.1542/peds.2018-0933 30705140

[2] Backes CH (2021). Proactive neonatal treatment at 22 weeks of gestation: a systematic review and meta-analysis. Am. J. Obstet. Gynecol..

[3] Ashmeade TL, Haubner L, Collins S, Miladinovic B, Fugate K (2016). Outcomes of a neonatal golden hour implementation project. Am. J. Med. Qual..

[4] Croop SEW, Thoyre SM, Aliaga S, McCaffrey MJ, Peter-Wohl S (2020). The golden hour: a quality improvement initiative for extremely premature infants in the neonatal intensive care unit. J. Perinatol..

[5] Wyckoff MH (2020). Neonatal life support 2020 international consensus on cardiopulmonary resuscitation and emergency cardiovascular care science with treatment recommendations. Resuscitation.

[6] Tay VY (2019). Admission temperature and hospital outcomes in extremely preterm infants. J. Paediatr. Child Health.

[7] Shah V, Hodgson K, Seshia M, Dunn M, Schmölzer GM (2018). Golden hour management practices for infants<32 weeks gestational age in Canada. J. Paediatr. Child Health.

[8] Hodgson KA, Owen LS, Lui K, Shah V (2021). Neonatal golden hour: a survey of Australian and New Zealand Neonatal Network Units’ early stabilisation practices for very preterm infants. J. Paediatr. Child Health.

[9] Mishra U (2021). Skincare practices in extremely premature infants: a survey of tertiary neonatal intensive care units from Australia and New Zealand. J. Paediatr. Child Health.

[10] Wan XL (2018). [Effect of golden-hour body temperature bundle management on admission temperature and clinical outcome in preterm infants after birth]. Zhongguo Dang Dai Er Ke Za Zhi.

[11] Sharma D (2020). Association between admission temperature and mortality and major morbidity in very low birth weight neonates–single center prospective observational study. J. Matern. Fetal Neonatal Med..

[12] Madar J (2021). European Resuscitation Council Guidelines 2021: newborn resuscitation and support of transition of infants at birth. Resuscitation.

[13] McCall, E. M., Alderdice, F., Halliday, H. L., Vohra, S. & Johnston, L. Interventions to prevent hypothermia at birth in preterm and/or low birth weight infants. *Cochrane Database Syst. Rev.* **2**, CD004210 (2018). 10.1002/14651858.CD004210.pub5 PMC6491068 29431872

[14] Jia Y (2013). Effect of delivery room temperature on the admission temperature of premature infants: a randomized controlled trial. J. Perinatol..

[15] Basu R, Malig B, Ostro B (2010). High ambient temperature and the risk of preterm delivery. Am. J. Epidemiol..

[16] Joseph RA, Derstine S, Killian M, Gephart S (2017). Ideal site for skin temperature probe placement on infants in the NICU. Adv. Neonat. Care.

[17] Bensouda B (2018). Temperature probe placement during preterm infant resuscitation: a randomised trial. Neonatology.

[18] Visscher M, Narendran V (2014). Neonatal infant skin: development, structure and function. Newborn Infant Nurs. Rev..

[19] Ågren, J., Segar, J. L., Söderström, F. & Bell, E. F. Fluid management considerations in extremely preterm infants born at 22-24 weeks of gestation. *Semin. Perinatol*. **46**, 151541 (2022). 10.1016/j.semperi.2021.151541 34848064

[20] Sinclair L, Crisp J, Sinn J (2009). Variability in incubator humidity practices in the management of preterm infants. J. Paediatr. Child Health.

[21] Kennedy PJ, Leathley CM, Hughes CF (2010). Clinical practice variation. Med J. Aust..

[22] Arbour, K. et al. Shifting provider attitudes and institutional resources surrounding resuscitation at the limit of gestational viability. *Am. J. Perinatol.* **39**, 869–877 (2022). 10.1055/s-0040-1719071 33111279

[23] Trevisanuto D (2022). Neonatal Resuscitation Practices in Europe: a survey of the Union of European Neonatal and Perinatal Societies. Neonatology.

