# Supplementary material for: Thermoregulation and golden hour practices in extremely preterm infants: an international survey
Source: Pediatr Res. 2022 Sep 8;93(6):1701–9. doi: 10.1038/s41390-022-02297-0 (PMC9453708; doi:10.1038/s41390-022-02297-0)
Supplement: Supplementary file 1 — Online supplementary material [file 41390_2022_2297_MOESM1_ESM.pdf]

Skin care practices in extremely premature infants: A survey of national and international tertiary neonatal intensive care units

I agree to participate

◯ Yes
◯ No

Country	__________________________________
State	__________________________________
The designation of the staff completing the survey on behalf of the NICU	◯ Medical<br>◯ Nursing
What is the lowest gestational age to whom your unit would offer resuscitation? (select one)	◯ 22 weeks<br>◯ 23 weeks<br>◯ 24 weeks<br>◯ 25 weeks
Approximately how many preterm infants born less than 28 weeks are admitted to your unit annually?	__________________________________

Following questions are related to birthing environment or resuscitation

Do you provide heated humidified gases at resuscitation ?	◯ Yes<br>◯ No<br>◯ Under consideration<br>◯ Not applicable
How does your unit transport preterm infants from birthing place to NICU?	☐ T-piece resuscitator CPAP/IPPV<br>☐ Dedicated transport ventilator<br>☐ Others<br>☐ Not applicable
Please specify	__________________________
What method does your unit use to minimise insensible water loss in the birthing environment?	☐ Ambient humidity<br>☐ Heated humidified gases at birth<br>☐ Bubble plastic<br>☐ Polyethylene occlusive plastic wraps<br>☐ Warm gel mattress or Heated mattress<br>☐ Other Commercial products<br>☐ Not applicable
Please specify	__________________________
How does your unit secure the skin temperature probe?	☐ Transparent adhesive dressings (e.g. Tegaderm)<br>☐ Hydrocolloids (e.g. Duoderm)<br>☐ Silicone based tapes (e.g. Siltape or Mepitac)<br>☐ Hydrogel based covers (e.g. reflective probe cover) Combination of above<br>☐ Any other product<br>☐ Not applicable
Please specify	__________________________
Where is the skin temperature probe secured?	◯ Axillae<br>◯ Front of abdomen<br>◯ On the back<br>◯ Any other site<br>◯ Not applicable
Specify	__________________________
What heating mode is used during transport?	◯ Manual<br>◯ Servo control<br>◯ Not applicable
Are you able to change the environmental ambient temperature at birthing place (birthing unit or operation theatres)?	◯ Yes<br>◯ No<br>◯ Under development<br>◯ Not applicable
What is the usual ambient temperature for the birthing place?	__________________________

Following questions are related to your unit practice on admission

As per your unit's current practice, please select what is routinely performed upon admission of preterm infants during Golden hour?

- ☐ Complete Anthropometry (weight, length, head circumference)
- ☐ Weight measurement only
- ☐ Intramuscular vitamin K injection
- ☐ Hepatitis B vaccine
- ☐ Heel prick for blood sugar&/capillary blood gas
- ☐ Commence humidification
- ☐ ECG lead application
- ☐ Non invasive blood pressure cuff application
- ☐ Pulse oxymetry
- ☐ Transcutanous monitoring if ventilated
- ☐ Cerebral oximetry using near infra-red spectroscopy
- ☐ Peripheral intra-venous cannulation
- ☐ Umbilical line insertion
- ☐ Chest x-ray
- ☐ Infection control surveillance cultures from skin (MRSA/ESBL)
- ☐ Other monitoring or intervention performed

Please specify

____

How soon after admission is humidity commenced?

- ◯ Within 60 mins
- ◯ Between 1-3hrs
- ◯ Between 3-6hrs
- ◯ Beyond 6hrs
- ◯ Not applicable

At what level is humidity commenced?

- ◯ 60-70 %
- ◯ 71-80 %
- ◯ 81-90 %
- ◯ >90 %
- ◯ Not applicable

At what age (in days) do you routinely start weaning humidity?

- ◯ < 3
- ◯ 3-7
- ◯ 8-14
- ◯ >15
- ◯ Not applicable

Does your unit have local guidelines on

	Yes	No	Not sure
Thermoregulation	○	○	○
Small baby protocol	○	○	○
Use of ambient humidity	○	○	○
	○	○	○
	○	○	○
	○	○	○
	○	○	○

☐
☐
☐
☐
☐

○
○
○
○
○
○

○
○

○
○
○
○

○
○

☐
☐
☐
☐
☐
☐
☐

Online supplementary material: Country, geographic region, and income status of the respondent units.

Number	Country	Geographic region	Income status
1	Afghanistan	Asia	Low
2	Albania	Europe	Upper-Middle
3	Argentina	South America	Upper-Middle
4	Australia	Oceania	High
5	Austria	Europe	High
6	Azerbaijan	Europe	Upper-Middle
7	Bahrain	Asia	High
8	Bangladesh	Asia	Lower-Middle
9	Belarus	Europe	Upper-Middle
10	Belgium	Europe	High
11	Bhutan	Asia	Lower-Middle
12	Bosnia and Herzegovina	Europe	Upper-Middle
13	Botswana	Africa	Upper-Middle
14	Brazil	South America	Upper-Middle
15	Bulgaria	Europe	Upper-Middle
16	Cameroon	Africa	Lower-Middle
17	Canada	North America	High
18	Chile	South America	High
19	China	Asia	Upper-Middle
20	Croatia	Europe	High
21	Czech Republic	Europe	High
22	Denmark	Europe	High
23	Dominican Republic	North America	Upper-Middle
24	Ecuador	South America	Upper-Middle
25	Egypt	Africa	Lower-Middle
26	Estonia	Europe	High
27	Ethiopia	Africa	Low
28	Finland	Europe	High
29	France	Europe	High
30	Germany	Europe	High
31	Ghana	Africa	Lower-Middle
32	Greece	Europe	High
33	Guatemala	North America	Upper-Middle
34	Honduras	North America	Lower-Middle
35	Hong Kong	Asia	High
36	Hungary	Europe	High
37	India	Asia	Lower-Middle
38	Indonesia	Asia	Lower-Middle
39	Iran	Asia	Lower-Middle
40	Ireland	Europe	High
41	Israel	Asia	High
42	Italy	Europe	High
43	Japan	Asia	High

44	Jordan	Asia	Upper-Middle
45	Kenya	Africa	Lower-Middle
46	Kuwait	Asia	High
47	Latvia	Europe	High
48	Lebanon	Asia	Upper-Middle
49	Lithuania	Europe	High
50	Malawi	Africa	Low
51	Malaysia	Asia	Upper-Middle
52	Malta	Europe	High
53	Mexico	North America	Upper-Middle
54	Moldova	Europe	Upper-Middle
55	Mongolia	Asia	Lower-Middle
56	Montenegro	Europe	Upper-Middle
57	Morocco	Africa	Lower-Middle
58	Nepal	Asia	Lower-Middle
59	Netherlands	Europe	High
60	New Caledonia	Oceania	High
61	New Zealand	Oceania	High
62	Nigeria	Africa	Lower-Middle
63	Norway	Europe	High
64	Oman	Asia	High
65	Pakistan	Asia	Lower-Middle
66	Panama	North America	Upper-Middle
67	Papua New Guinea	Oceania	Lower-Middle
68	Paraguay	South America	Upper-Middle
69	Philippines	Asia	Lower-Middle
70	Poland	Europe	High
71	Portugal	Europe	High
72	Qatar	Asia	High
73	Romania	Europe	Upper-Middle
74	Russia	Asia	Upper-Middle
75	Rwanda	Africa	Low
76	Saudi Arabia	Asia	High
77	Serbia	Europe	Upper-Middle
78	Singapore	Asia	High
79	Slovakia	Europe	High
80	Slovenia	Europe	High
81	South Africa	Africa	Upper-Middle
82	South Korea	Asia	High
83	Spain	Europe	High
84	Sri Lanka	Asia	Lower-Middle
85	Sweden	Europe	High
86	Switzerland	Europe	High
87	Taiwan	Asia	High
88	Tanzania	Africa	Lower-Middle
89	Thailand	Asia	Upper-Middle
90	Turkey	Europe	Upper-Middle

91	UK (England, Wales, Scotland, Northern Ireland)	Europe	High
92	Ukraine	Europe	Lower-Middle
93	Uruguay	South America	High
94	USA	North America	High
95	Vietnam	Asia	Lower-Middle
96	Zambia	Africa	Lower-Middle
97	Zimbabwe	Africa	Lower-Middle

Source: www.worldbank.com (2021)
